# Interdisciplinary Educational Interventions Improve Knowledge of Eating, Nutrition, and Physical Activity of Elementary Students

**DOI:** 10.3390/nu14142827

**Published:** 2022-07-09

**Authors:** Mayra Lopes de Oliveira, Juliana de Lara Castagnoli, Kerulyn Maria Chanivski Machado, Jaqueline Machado Soares, Flávia Teixeira, Dalton Luiz Schiessel, Elisvânia Freitas dos Santos, Daiana Novello

**Affiliations:** 1 Postgraduate Program Interdisciplinary in Community Development, Department of Nutrition, State University of Midwest, Guarapuava 85040-167, Brazil; mayra.lopes2010@gmail.com (M.L.d.O.); julara2008@hotmail.com (J.d.L.C.); kerulyn.maria1@outlook.com (K.M.C.M.); jaquue.s@gmail.com (J.M.S.); teixeiraflavia19@gmail.com (F.T.); 2 Department of Nutrition, State University of Midwest, Guarapuava 85040-167, Brazil; daltonls68@gmail.com; 3 Postgraduate Program in Biotechnology, Faculty of Pharmaceutical Sciences, Food and Nutrition, Federal University of Mato Grosso do Sul, Campo Grande 79070-900, Brazil; elisvania@gmail.com

**Keywords:** children, health education, health promotion

## Abstract

This research aimed to evaluate the interdisciplinary educational intervention effects on knowledge of eating, nutrition, and physical activity in elementary-school students. Participants were 368 school children enrolled in public schools. The research was organized in three stages: pre-intervention, intervention and post-intervention. In pre-intervention, children were evaluated regarding their nutritional status. They also answered questionnaires related to eating and nutrition and physical activity. In the intervention stage, educational interventions were carried out on the same topics for a period of five months; in post-intervention, children answered the same questionnaires applied in pre-intervention. Despite most children having normal nutritional status (58.2%), a high number of students were overweight (38%). In the initial phase, it was found that most children had excellent knowledge of eating, nutrition, and good physical activity knowledge (*p*-value < 0.05). Educational health intervention significantly increased children’s knowledge of eating, nutrition, and physical activity, when evaluated in the post-intervention period. Both boys and girls increased their knowledge of eating, nutrition, and physical activity after the application of interdisciplinary interventions (*p*-value < 0.05). A similar effect was observed for children with different nutritional status. It is concluded that interdisciplinary educational interventions carried out for children in an elementary-school environment are effective for improving knowledge of eating, nutrition, and in physical activity, promoting healthier habits among children.

## 1. Introduction

Obesity results in an energy imbalance between caloric food consumption and caloric expenditure. Currently, more than 650 million adults are obese, representing 13% of the global population. In relation to children, it is estimated that 340 million children aged between 5 and 19 years old and 40 million children under 5 years old are overweight [1]. In Brazil, overweight and obesity has increased by 239% in 20 years, with 15% of school children and adolescents (6 to 18 years old) being diagnosed with overweight and 5% with obesity [2].

Childhood obesity has immediate and long-term consequences for health in addition to strongly predicting nutritional status in adulthood [1]. One of the factors that contributes to increased obesity worldwide prevalence is the nutritional transition process. This process is determined by systemic social changes, such as increased industrialization, urbanization and improving economic conditions. Prior to the nutritional transition phase, food was mainly based on legumes, fruit, vegetables and grains. However, over the years, there has been an increasing consumption of processed and ultra-processed foods, with a high content of fat, sugar and energy, such as fast food. In addition, there was a reduction in physical activity practice [3], since children prefer the use of electronic devices to the practice of physical activities [4]. Likewise, the use of less active transportation facilities [5], such as cars and buses, to move around, and additionally the reduction in recreational places for physical exercise practice, supports a sedentary lifestyle [6].

Habits related to food and physical activity are the main factors that influence human health from childhood [7]. Food consumption based on healthy foods, such as fruit and vegetables, for example, together with the regular physical activity, can reduce the risk of chronic non-communicable diseases, such as obesity and diabetes [8]. In addition, they can collaborate to reduce anxiety [9], depression, and increase self-esteem, cognitive thinking [10], socialization and academic performance [11]. School children have an average fruit and vegetable consumption of 236 g/day [12], well short of the daily recommendation of five servings, equivalent to 400 g as determined by the World Health Organization (WHO) [13]. On the other hand, beverages and foods with high sugar content, such as sweetened drinks, stuffed cookies and candies, are the most consumed by this section of the public [14]. Similar effects are observed for physical activity practice, since less than 10% of children perform 60 min of moderate to vigorous physical activity per day [15], which is the current recommendation [16]. In addition, physical exercise levels may be even lower in adulthood when compared to childhood and adolescence [15]. In Brazil, the proportion of children who comply with physical activity guidelines is higher, especially in the southeast (44.1%) [17] and the south (53.3%) [18].

Some factors have a direct influence on children’s food consumption, such as socializing with friends and teachers [19], and parents’ and/or caregivers’ beliefs and attitudes towards food, which may include emotional and cultural aspects [20], knowledge in nutrition and socioeconomic conditions [21]. Likewise, the media [22], gender and urbanization [23] can affect physical activity practice. Despite this scenario, it is essential that children receive guidance on the importance of consuming healthy foods and periodic physical exercise, which can promote good health habits in subsequent stages of life, reducing the risk of disease [24].

Health education aims to share information to the community, groups or individuals. In the case of activities related to nutrition education aimed at children, it has already been proven that theoretical activities for a prolonged period of time have positive effects on knowledge and food attitudes [25]. The use of practical activities that include exercises promotes an increase in physical activity levels [26]. However, studies that use joint techniques of nutrition and physical activity obtain even more effective results, such as a significant increase in fruit and vegetable consumption, reduction of fried foods and fast foods consumption, and excess weight reduction [27,28]. In this context, the objective of this research was to evaluate the interdisciplinary educational intervention effect on the knowledge of eating, nutrition, and physical activity of elementary-school-aged children.

## 2. Materials and Methods

### 2.1. Study Design

**Figure 1:**
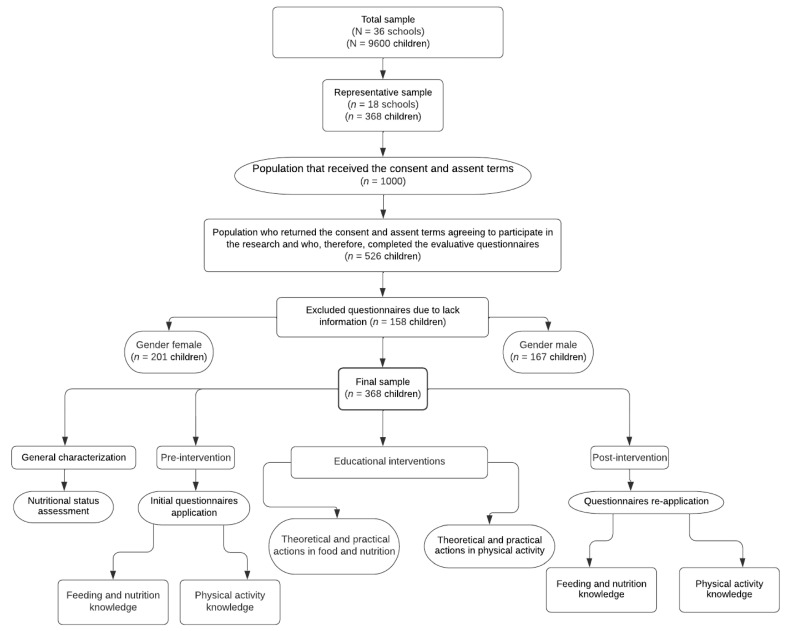
Research steps flowchart carried out with school-aged children.

This is a descriptive, prospective, cross-sectional study starting in 2019 and ending in 2021, carried out in Guarapuava city, Parana state, Brazil. School-aged children enrolled in public schools participated in the research. Initially, nutritional status was evaluated. Then, in the pre-intervention stage, children answered questionnaires related to eating, nutrition, and physical activity. Subsequently, in the intervention stage, educational actions were carried out on the same topics for a period of five months. Finally, in the post-intervention stage, participants answered the same questionnaires applied during pre-intervention, which allowed the assessment of the children’s learning during the study. All research stages were developed in a joint and interdisciplinary manner, involving professionals and academics in the fields of nutrition and physical education. Figure 1 shows a detailed study stages flowchart.

### 2.2. Population

Research was carried with a representative sample of total number of children (9600) of school age (7–10 years), enrolled between the 2nd and 5th grades in 36 public schools in an urban area. Sample determination was carried out in two stages: (1) the schools were selected by means of non-probabilistic sampling for convenience, the one with the largest students number being chosen, thus totaling 18 schools; (2) children were chosen through simple random sampling, considering the following parameters—total number of students enrolled in the 2nd to 5th grades of urban schools in the city, a confidence level of 95% and maximum error of 5%, totaling a minimum representative sample of 368 students.

### 2.3. Assessment of Nutritional Status

Anthropometric data collection from children was performed by previously trained registered dietitians. Weight (kg) was obtained using a portable digital scale (Tanita^®^, Arlington Heights, IL, USA), with precision of 100 g, while height (m) was verified using an inelastic measuring tape (100 cm, precision of 0.1 cm) fixed to the wall (without plinth) [29]. To calculate Body Mass Index (BMI), the following formula was used:

*weight*/*height*^2^

Results were expressed as percentile values in relation to a reference population median, using BMI-for-age cut-off points for Brazilian children aged 5 to 10 years, adapted from the World Health Organization (WHO) [29]. Nutritional status diagnosis was evaluated considering children together and separated by gender, according to the following classifications: “severe thinness” (<0.1st percentile); “thinness” (≥0.1st percentile and <3rd percentile); “eutrophy” (≥3rd percentile and ≤85th percentile); “overweight” (>85th percentile and ≤97th percentile); “obesity” (>97th percentile and ≤99.9th percentile); “severe obesity” (>99.9th percentile). For purposes of statistical comparison, nutritional status was also categorized as follows: (a) “low weight” (severe thinness and thinness), <3rd percentile; (b) “eutrophy” (adequate nutritional status), ≥3rd percentile and ≤85th percentile; (c) “overweight” (overweight, obesity and severe obesity), >85th percentile. The category “eutrophy” was adopted as the reference group.

### 2.4. Pre-Intervention Stage

At this stage, children answered two questionnaires, one about knowledge about eating and nutrition and other about knowledge about physical activity.

#### 2.4.1. Eating and Nutrition Knowledge

Eating and nutrition questions (ENQ) were based on content presented in the Food Pyramid Guide [30]. Thus, it aimed to verify knowledge about food groups. The questionnaire consisted of four general illustrative multiple-choice questions belonging to food pyramid groups: Question 1 (ENQ 1)—“Which foods have more carbohydrate?”; Question 2 (ENQ 2)—“Which foods have enough vitamins and minerals?”; Question 3 (ENQ 3)—“Which foods have more protein?”; Question 4 (ENQ 4)—“Which foods have a lot of sugar and/or fat?”. Each question had eight alternatives, of which only four were correct. Children were classified as follows: “low knowledge” (0 to 5 points), “good knowledge” (6 to 10 points) and “great knowledge” (11 to 16 points) [31]. The eating and nutrition knowledge questionnaire showed satisfactory internal validity (Cronbach’s alpha, α = 0.74) [32].

#### 2.4.2. Physical Activity Knowledge

An instrument on knowledge of physical activity, with an approach to exercising importance for health, was developed based on questionnaires by Domingues et al. [33] and Silveira and Silva [34]. Its purpose was to investigate children’s understanding of physical activity benefits for health, prevention of chronic disease and their effects on human body. The Physical Activity Questions (PAQ) consisted of a questionnaire with four general illustrative multiple-choice questions: Question 1 (PAQ 1)—“Physical activity performed daily prevents which these diseases?”; Question 2 (PAQ 2)—“Can exercise lack leads a person to have?”; Question 3 (PAQ 3)—“What the practice physical activity improves on a daily basis?”; Question 4 (PAQ 4)—“What activities can help you to have good health?”. Each question had eight alternatives, of which only four were correct. Children were classified as follows: “low knowledge” (0 to 5 points), “good knowledge” (6 to 10 points) and “great knowledge” (11 to 16 points) [31]. The physical activity knowledge questionnaire showed very good internal validity (Cronbach’s alpha, α = 0.81) [32].

### 2.5. Intervention Stage

Health education activities related to eating, nutrition, and physical activity topics were applied in an interdisciplinary way by professionals and academics in nutrition and physical education areas. Interventions were carried out for five months, with two meetings per month, totaling ten meetings per school. Each intervention lasted a total of 30 min. The first 15 min were used to explain the topics covered, then expository and participatory activities were carried out. The exercises were carried out in interspersed and individual meetings, with respect to each knowledge area. After the theoretical explanation, a practical dynamic was applied, reinforcing theoretical knowledge. Activities were aimed at providing students with an identification and reflection of problems, raising hypotheses and pointing out solutions to a reduce sedentary lifestyle. In this way, educational and dynamic lectures were included, using simple language, which helps with fixing and understanding the content. Throughout the educational process, active participation and interaction with students was prioritized.

#### 2.5.1. Eating and Nutrition Actions

Recreational–educational activities relating to eating and nutrition were prepared according to the Food Pyramid Guide [30], which visually represents food concepts such as proportion, moderation and variety. Topics covered were food groups; food and its different functions in the body; and recommended daily portions [30]. This intervention aimed to build, with children’s help, the pyramid levels from previous explanations, encouraging the consumption of lower caloric foods and greater intake of foods with a high nutrient content, promoting a healthy and varied diet. All interventions related to eating and nutrition were carried out in classrooms and in the schoolyard, facilitating learning.

#### 2.5.2. Physical Activity Actions

Actions were based on content covered in guidelines [35,36] and manuals [37]. Materials included physical activity recommendations for children and adolescents, including information on quantity, types and intensity, health benefits, and encouragement for the daily practice of activities and games. In addition, topics related to occasional problems caused by physical activity were considered, especially a sedentary lifestyle in childhood and prolonged use of electronic devices, such as cell phones, computers, video games and television. Also, the outdoor children’s games practice that, rescued popular culture and body movement, such as running, jumping and walking, was encouraged. The intervention objective was to make students aware of regular exercise, both in a school environment and outside, in addition to promoting interactive activities between groups. Actions were carried out in specific places at school, such as the physical education court. Table 1 describes activities related to eating, nutrition, and physical activity carried out in schools.

### 2.6. Post-Intervention Stage

Questionnaires applied in pre-intervention stage were reapplied at this stage to assess the learning effect from educational actions (intervention stage) on knowledge about eating, nutrition, and physical activity.

### 2.7. Statistical Analysis

Results were evaluated using mean frequency and standard deviation, depending on the case. Nutritional assessment was performed using Epi Data program (Data Management and Basic Statistical Analysis System, version 3.1, Odense, Denmark). To perform the analyses, R software, version 4.0.3, was used, using descriptive procedures and inferential statistics. Nonparametric tests—Pearson’s and Wilcoxon’s chi-square tests—and parametric tests—paired Student’s t test and Tukey’s test—were used to evaluate data. A significance level of 5% (*p*-value < 0.05) was adopted for analyses.

### 2.8. Ethical Issues

Research was approved by the Ethics Committee for Research Involving Human Beings (COMEP) of UNICENTRO, under opinion No. 3089,447/2018.

## 3. Results and Discussion

### 3.1. Nutritional Status Assessment

**Figure 2:**
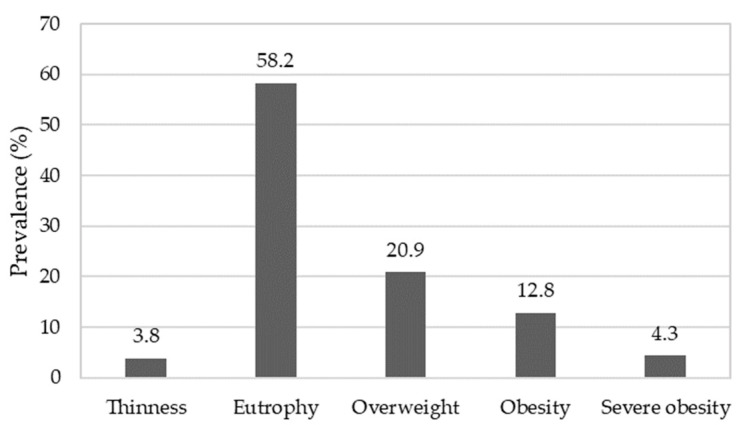
Elementary-school children nutritional status. Thinness: ≥0.1st percentile and <3rd percentile; eutrophy: ≥3rd percentile and ≤85th percentile; overweight: >85th percentile and ≤97th percentile; obesity: >97th percentile and ≤99.9th percentile; severe obesity: >99.9th percentile.

The children had a mean age of 8.65 ± 0.8 years, with 8.6 ± 0.8 years for girls and 8.7 ± 0.7 years for boys. The BMI mean was 18.3 ± 3.8 kg/m^2^, differing between girls (17.9 ± 3.3 kg/m^2^, eutrophy—≥3rd percentile and ≤85th percentile) and boys (18.7 ± 4.0 kg/m^2^, overweight—>85th percentile and ≤97th percentile), considering mean age for each gender. Figure 2 shows the participant nutritional status.

Most children presented eutrophic nutritional status. However, a high number of students were classified as overweight (38%) for age and gender [43], corroborating other studies carried out in China [44], the United States [45] and Italy [46]. In Brazil, similar results were observed in the northeast [47] and southeast [48]. In addition to genetic and behavioral factors, such as diets and sleep duration, socioeconomic factors, family environment and food preferences are also associated with the prevalence of childhood obesity [49]. The environment can also influence nutritional status, as demonstrated by Au et al. [50]. In addition, authors also assessed the quality of children’s food received at school and at home, using the US Department of Agriculture Food and Nutrient Database for Dietary Studies (version 3.0, USDA, Beltsville, MD, USA, 2008). They concluded that meals offered in a school environment had better overall quality compared to those offered at home. This fact can directly interfere with a child nutritional status. This reinforces the importance of expanded educational actions that promote healthier food consumption.

### 3.2. Evaluation of Pre- and Post-Intervention Stages

Table 2 shows the children’s response prevalence to questionnaires about their knowledge of eating, nutrition, and physical activity in the pre- and post-intervention stages.

Regarding the pre-intervention stage, most children had excellent knowledge of eating and nutrition and good physical activity knowledge (*p*-value < 0.05). A smaller percentage was classified as having low knowledge in both subjects. These results demonstrate that participants had some prior knowledge about eating, nutrition, and physical activity before receiving educational intervention. Schools and advertising media are increasingly concerned with educating children about the importance of physical activity and nutrition to prevent chronic diseases and improve quality of life [51].

**Figure 3:**
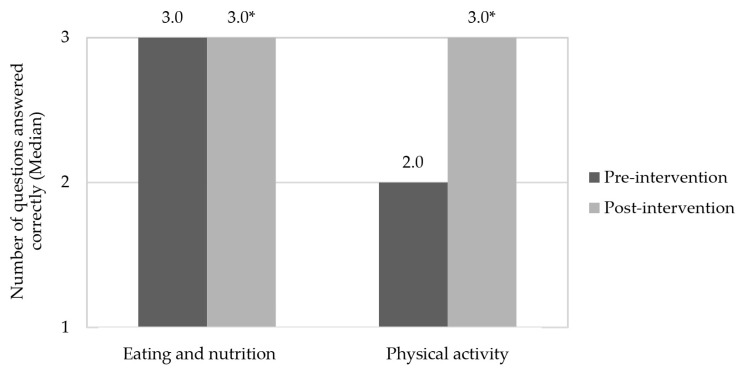
Children’s correct answers to questions in the questionnaires about knowledge of eating, nutrition, and physical activity in the pre- and post-intervention stages. * Indicates a significant difference by Wilcoxon Test (*p*-value < 0.05), in relation to the correct answers to questions of the questionnaires about knowledge in eating, nutrition, and physical activity.

After educational health actions (post-intervention), almost all children showed excellent knowledge both in eating, nutrition, and in physical activity, which demonstrates the effectiveness of this eating and nutrition education for this population, corroborating other studies [52,53]. In addition, recreational activities aimed at promoting health are considered a great opportunity to create relationships that favor sharing of knowledge and experiences, instructing the individual to take care of their own health [51]. According to Drapeau et al. [54], nutritional education can also improve healthy food consumption. Figure 3 shows the medians of correct responses for children in the pre-intervention and post-intervention periods, in relation to the knowledge of eating and nutrition, and physical activity, respectively.

Health education activities carried out during the intervention stage increased children’s knowledge of eating, nutrition, and physical activity in the post-intervention period (*p*-value < 0.05). Similar effects were observed by Franciscato et al. [53] and Syrmpas et al. [55] after interventions carried out in schools with children in Brazil and Greece, respectively. In this respect, the school environment is ideal for carrying out preventive health education actions due to its structure, effectiveness and wide coverage of individuals [56]. In addition, studies have already shown that these interventions can positively influence fruit and vegetable consumption, nutritional knowledge, energy and sugar intake [57] and physical activity [26].

**Figure 4:**
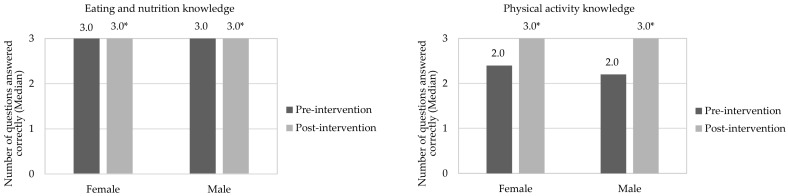
Correct answers to questions about knowledge of eating, nutrition, and physical activity in the pre- and post-intervention stages, considering the children’s genders. * Indicates significant difference by Wilcoxon Test (*p*-value < 0.05), in relation for correct answers to questions of the questionnaires about knowledge in eating, nutrition, and physical activity in the pre- and post-intervention stages. The assessment was performed between the same gender. Grade scale: 1—low, 2—good, and 3—great.

**Figure 5:**
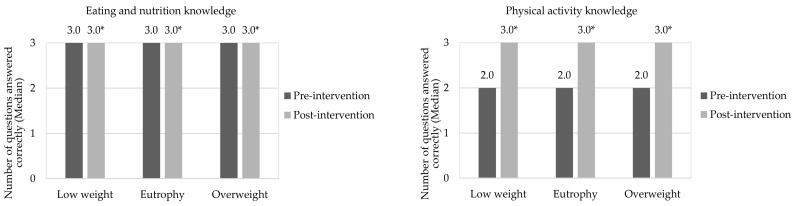
Correct answers to questions about knowledge of eating, nutrition, and physical activity in the pre- and post-intervention stages, considering the children’s nutritional statuses. * Indicates significant difference by Wilcoxon Test (*p*-value < 0.05), in relation for correct answers to questions of the questionnaires about knowledge in eating, nutrition, and physical activity in the pre- and post-intervention stages. The assessment was performed between the same nutritional status. Grade scale: 1—low, 2—good, and 3—great.

Figure 4 and Figure 5 show the median correct responses for children in relation to eating, nutrition, and physical activity in pre- and post-intervention stages, separated by gender and nutritional status, respectively. Both boys and girls increased their knowledge of eating, nutrition, and physical activity after interdisciplinary intervention application (*p*-value < 0.05). A similar effect was observed when children were evaluated in nutritional status terms, which was not observed in the study by Franciscato et al. [53]. It is noteworthy that nutritional knowledge may not be directly related to actual dietary practices. Thus, despite children having adequate knowledge about the effects of unhealthy diets on health, they continue to consume them [58]. In this aspect, it is possible that the effective change in eating habits is influenced by other factors, such as family environment [59], advertising [60], the socioeconomic conditions of those responsible [21] and children’s interaction with friends and teachers [19]. The same occurs with physical activity practice, since awareness of its importance can promote positive change in behavior in relation to physical activity in this age group [26].

Children’s knowledge level regarding eating, nutrition, and physical activity in pre- and post-intervention stages, considering gender and nutritional status, can be seen in Table 3. Evaluating the knowledge topic in eating and nutrition in the pre-intervention stage, it is observed that most female and male children with nutritional status of eutrophic and overweight presented excellent knowledge. Few participants (≤7%) were assessed with low knowledge. Children’s responses with low weight showed no difference between good and excellent knowledge (*p*-value > 0.05), and none were classified as having low knowledge. Regarding physical activity, most underweight and eutrophic boys had good knowledge, while girls and overweight children were considered to have excellent knowledge (*p*-value < 0.05). Similar to what happened in the topic of eating and nutrition, a lower children number, regardless of gender and nutritional status, were classified as having low physical activity knowledge relating to health (≤18%).

At post-intervention stage, excellent knowledge of eating and nutrition, regardless of gender and nutritional status (*p*-value < 0.05), was observed for most children. It is noteworthy that a very low number of participants (≤0.7%) had good and low knowledge in this topic. Additionally, all underweight children had excellent knowledge. As for the approach to physical activity, most children had excellent knowledge, except for one female and one with low weight who were classified as having good knowledge. In this context, playful educational interventions aimed at health can help in the learning process, relating theory to practice [55]. Furthermore, interdisciplinary educational actions improved children’s responses in terms of excellent knowledge (>90%) on topics of eating, nutrition, and physical activity (Table 3), an effect also observed in research by Fuller et al. [61] with Danish children.

**Figure 6:**
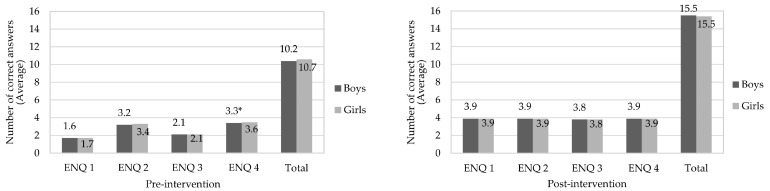
Average of correct answers to questions about eating and nutrition knowledge in the pre- and post-intervention stages, compared between genders. * Means indicate significant difference by Student’s t test (*p*-value < 0.05) in the same question. Eating and Nutrition Questions (ENQ): ENQ 1—“Which foods have more carbohydrate?”; ENQ 2—“Which foods have enough vitamins and minerals?”; ENQ 3—“Which foods have more protein?”; ENQ 4—“Which foods have a lot of sugar and/or fat?”; Each question contained 4 correct answers; for total there were 16 correct answers.

**Figure 7:**
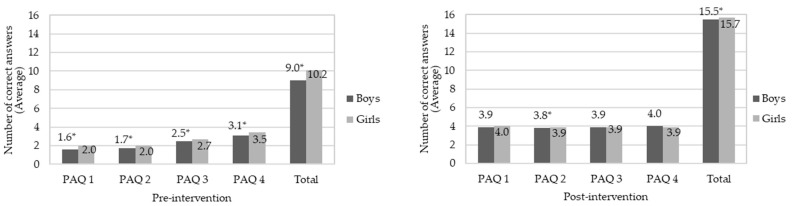
Average of correct answers to questions about knowledge about physical activity in pre- and post-intervention stages, compared between genders. * Means indicate significant difference by Student’s t test (*p*-value < 0.05) in the same question. Physical Activity Questions (PAQ): PAQ 1—“Physical activity performed daily prevents which these diseases?”; PAQ 2—“Can exercise lack leads a person to have?”; PAQ 3—“What the practice physical activity improves on a daily basis?”; PAQ 4—“What activities can help you to have good health?”; Each question contained 4 correct answers; for the total there were 16 correct answers.

Figure 6 and Figure 7 show correct answer averages to questions about eating and nutrition knowledge in pre- and post-intervention stages, compared between genders. Regarding the pre-intervention stage, boys were less assertive than girls (*p*-value < 0.05) for sugar and/or fat group (ENQ 4). At the post-intervention stage, there was no significant difference between genders for all questions. As for knowledge about physical activity (pre-intervention), boys scored less correctly than girls in all questions (*p*-value < 0.05). A similar effect occurred after intervention for questions PAQ 2 and in total assessment of responses; however, there was no significant difference (*p*-value > 0.05) to questions PAQ 1, PAQ 3 and PAQ 4. According to Jalkanen et al. [62], girls are generally more concerned with their body weight. In addition, they suffer social and family influences and demands for a thin body, factors that may explain the greater female knowledge of topics addressed in this research.

Table 4 shows children’s correct answer averages comparing pre- and post-intervention stages, in relation to knowledge of eating, nutrition, and physical activity. At the pre-intervention stage, ENQ 2, ENQ 4 and PAQ 4 questions were ones in which participants scored the most correct answers (*p*-value < 0.05), while ENQ 1, PAQ 1 and PAQ 2 questions had fewer correct answers (*p*-value < 0.05). Generally, children have greater preference and acceptance for foods with high sugar and fat levels [63], and less when it comes to fruit and vegetables [64]. They also have high knowledge level about the sugar content present in foods [60] and about the importance of fruit and vegetable consumption and their health effects [65]. However, their choices are based on availability and accessibility of food purchased by family members, advertising and preferences [60]. This context may explain children’s greater knowledge of these topics.

When individual answers at post-intervention stage are evaluated, it is verified that the proposed knowledge of foods with carbohydrate (ENQ 1), protein (ENQ 3), what lack of exercise can cause (PAQ 2) and what are the benefits of daily physical activity practice (PAQ 3) were the questions that had less effectiveness in children’s learning (*p*-value < 0.05). Despite this, educational activities in eating, nutrition, and physical activity increased children’s knowledge of all questions (*p*-value < 0.05), which are corroborated by the literature [52].

Considering the positive effects observed by interdisciplinary interventions applied in the present research, it can be inferred that results can contribute to increase food consumption with favorable nutritional profile in childhood. Thus, it corroborates the WHO [66], which recommends health education for children, guardians and teachers, including the importance of consuming healthy foods and reducing sugar and fat intake, in addition to promoting physical exercise. In Brazil, this topic is regulated by the National School Eating Program (Programa Nacional de Alimentação Escolar—PNAE), which aims to meet the nutritional needs of children at school and form healthy eating habits through food and nutrition education [67]. Another piece of legislation deals with the School Health Program (Programa Saúde na Escola—PSE), which aims to contribute to students wellbeing through actions of promotion, prevention and healthcare [68].

## 4. Conclusions

Eutrophic nutritional status was verified for most school-aged children; however, a high number of these children are overweight for their age. In general, children have good knowledge of topics related to eating, nutrition, and physical activity. This knowledge is enhanced after carrying out educational health interventions, when applied in an interdisciplinary manner and for a prolonged period of time. This effect was observed for boys and girls and when children were organized by different nutritional status. However, boys stand out for having less knowledge, especially in specific subjects related to physical activity. Finally, it is concluded that educational health interventions applied in an interdisciplinary way in the school environment are effective for improving knowledge in eating, nutrition, and physical activity, promoting healthier habits among children.

## Figures and Tables

**Table 1:** Nutrition education and physical activity activities developed in schools.

Month	Activity	
	Theoretical	Practice
Eating and nutrition		
1	Cereal group	Food traffic light [38]
2	Fruit and vegetables group	Seasonality [39]
3	Dairy, meat and derivatives group	Healthy bowling play [40]
4	Beans and oilseeds group	Hangman play and beans word search [39]
5	Oils and fats and sugars and sweets groups	Amount of sugars and fats in foods [39]
Physical education		
1	Disease prevention through daily physical activity	Undead play [41]
2	Consequences of physical activity lack	Hopscotch play [41]
3	Daily physical activity benefits	Mirror play [41]
4	Physical exercises that help promote health	Musical chairs play [42]
5	Review of previously discussed topics	Capture the flag play [42]

**Table 2:** Children’s responses prevalence to questions about knowledge about eating, nutrition, and physical activity in the pre- and post-intervention stages.

	Knowledge Level						
Variable	Low		Good		Great		*p*-Value
	*n*	%	*n*	%	*n*	%	
Pre-intervention							
Eating and nutrition knowledge	22	6.0 ^c^	131	35.6 ^b^	215	58.4 ^a^	<0.001
Physical activity knowledge	51	13.9 ^c^	162	44.0 ^a^	155	42.1 ^b^	<0.001
Post-intervention							
Eating and nutrition knowledge	1	0.3 ^c^	2	0.5 ^b^	365	99.2 ^a^	<0.001
Physical activity knowledge	*	*	1	0.3 ^b^	367	99.7 ^a^	<0.001

^a,b,c^ Letters was refer to Chi-Square Test results; *n* = 368; * There was no response; Post hoc adjusted standardized residuals.

**Table 3:** Children’s knowledge level regarding eating, nutrition, and physical activity in the pre- and post-intervention stages, considering gender and nutritional status.

		Knowledge Level						
Variable		Low		Good		Great		*p*-Value
		*n*	%	*n*	%	*n*	%	
Eating and nutrition knowledge								
Pre-intervention	Gender							
	Female (*n* = 201)	13	6.5 ^c^	65	32.3 ^b^	123	61.2 ^a^	<0.001
	Male (*n* = 167)	9	5.4 ^c^	66	39.5 ^b^	92	55.1 ^a^	<0.001
	Nutritional status							
	Low weight (*n* = 14)	-	-	6	42.9 ^a^	8	57.1 ^a^	=0.593
	Eutrophy (*n* = 214)	15	7.0 ^c^	79	36.9 ^b^	120	56.1 ^a^	<0.001
	Overweight (*n* = 140)	7	5.0 ^c^	46	32.9 ^b^	87	62.1 ^a^	<0.001
	Physical activity knowledge							
	Gender							
	Female (*n* = 201)	21	10.4 ^c^	83	41.3 ^b^	97	48.3 ^a^	<0.001
	Male (*n* = 167)	30	18.0 ^c^	79	47.3 ^a^	58	34.7 ^b^	<0.001
	Nutritional status							
	Low weight (*n* = 14)	1	7.1 ^c^	9	64.3 ^a^	4	28.6 ^b^	=0.030
	Eutrophy (*n* = 214)	29	13.6 ^c^	100	46.7 ^a^	85	39.7 ^b^	<0.001
	Overweight (*n* = 140)	21	15.0 ^c^	53	37.9 ^b^	66	47.1 ^a^	<0.001
	Eating and nutrition knowledge							
Post-intervention	Gender							
	Female (*n* = 201)	1	0.5 ^b^	1	0.5 ^b^	199	99.0 ^a^	<0.001
	Male (*n* = 167)	*	*	1	0.6 ^b^	166	99.4 ^a^	<0.001
	Nutritional status							
	Low weight (*n* = 14)	*	*	*	*	14	100.0	-
	Eutrophy (*n* = 214)	1	0.5 ^b^	1	0.5 ^b^	212	99.1 ^a^	<0.001
	Overweight (*n* = 140)	*	*	1	0.7 ^b^	139	99.3 ^a^	<0.001
	Physical activity knowledge							
	Gender							
	Female (*n* = 201)	*	*	1	0.5 ^b^	200	99.5 ^a^	<0.001
	Male (*n* = 167)	*	*	*	*	167	100.0	-
	Nutritional status							
	Low weight (*n* = 14)	*	*	1	7.1 ^b^	13	92.9 ^a^	<0.001
	Eutrophy (*n* = 214)	*	*	*	*	214	100.0	-
	Overweight (*n* = 140)	*	*	*	*	140	100.0	-

^a,b,c^ Letters was refer to Chi-Square Test results; *n* = 368; * There was no response; Adjusted standardized residuals post-hoc.

**Table 4:** Average of correct answers (±standard deviation) of questions about knowledge about eating, nutrition, and physical activity comparing pre-intervention and post-intervention stages.

Question	Pre-Intervention	Post-Intervention	*p*-Value
	Number of Correct Answers (Average ± SD)	Number of Correct Answers (Average ± SD)	
Eating and nutrition knowledge			
ENQ 1	1.67 ± 1.1 ^c^	3.86 ± 0.5 ^b, c^	<0.01
ENQ 2	3.28 ± 1.1 ^a^	3.93 ± 0.3 ^a, b^	<0.01
ENQ 3	2.11 ± 1.0 ^b^	3.79 ± 0.6 ^c^	<0.01
ENQ 4	3.45 ± 0.9 ^a^	3.94 ± 0.3 ^a^	<0.01
Total hits	10.50 ± 2.8	15.49 ± 1.3	<0.01
Physical activity knowledge			
PAQ 1	1.80 ± 1.3 ^c^	3.94 ± 0.3 ^a, b^	<0.01
PAQ 2	1.89 ± 1.2 ^c^	3.87 ± 0.4 ^c^	<0.01
PAQ 3	2.61 ± 1.1 ^b^	3.88 ± 0.3 ^b, c^	<0.01
PAQ 4	3.30 ± 1.1 ^a^	3.95 ± 0.3 ^a^	<0.01
Total hits	9.63 ± 3.6	15.63 ± 0.9	<0.01

^a,b,c^*p*-value < 0.05 indicates significant difference between the average in line by Student’s *t* test for paired samples; different letters at the column indicate significant difference by Tukey’s test (*p*-value < 0.05); Eating and Nutrition Questions (ENQ): ENQ 1—“Which foods have more carbohydrate?”; ENQ 2—“Which foods have enough vitamins and minerals?”; ENQ 3—“Which foods have more protein?”; ENQ 4—“Which foods have a lot of sugar and/or fat?”; Physical Activity Questions (PAQ): PAQ 1—“Physical activity performed daily prevents which these diseases?”; PAQ 2—“Can exercise lack leads a person to have?”; PAQ 3—“What the practice physical activity improves on a daily basis?”; PAQ 4—“What activities can help you to have good health?”.

## Data Availability

The data presented in this study are available on request from the corresponding author. The data are not publicly available due to privacy restrictions.
